# Characterization of reactive stroma in prostate cancer: involvement of growth factors, metalloproteinase matrix, sexual hormones receptors and prostatic stem cells

**DOI:** 10.1590/S1677-5538.IBJU.2014.0355

**Published:** 2015

**Authors:** Maurício Moreira da Silva, Wagner Eduardo Matheus, Patrick Vianna Garcia, Rafael Mamprim Stopiglia, Athanase Billis, Ubirajara Ferreira, Wagner José Fávaro

**Affiliations:** 1 Departamento de Cirurgia, Área de Urooncologia, Faculdade de Ciências Médicas, Universidade Estadual de Campinas (UNICAMP), Campinas, SP, Brasil; 2 Laboratório de Carcinogênese Urogenital e Imunoterapia (LCURGIM), Departamento de Biologia Estrutural e Funcional, Universidade Estadual de Campinas (UNICAMP), Campinas, SP, Brasil; 3 Departamento de Patologia, Faculdade de Ciências Médicas, Universidade Estadual de Campinas (UNICAMP), Campinas, SP, Brasil

**Keywords:** Reactive stroma, prostate cancer, growth factors, sexual hormones receptors, epithelium-stroma interaction

## Abstract

**Introduction and Objectives:**

Reactive Stroma (RStr) is observed in many human cancers and is related to carcinogenesis. The objectives of the present study were to stablish a relationship of the RStr microenvironment with prostate cancer (Pca) through a morphological and molecular characterization, and to identify a possible relationship between RStr with worse prognosis factors and occurrence of malignant prostatic stem cells.

**Materials and Methods:**

Forty prostatic samples were selected from men with Pca diagnosis submitted to radical prostatectomy; they were divided in two groups: Group-1 (n=20): samples without reactive stroma; Group-2 (n=20): samples of PCa with intense stroma reaction. Prostatic samples were evaluated for RStr intensity by Masson Trichromic stain and posteriorly submitted to histopathological and immunohistochemistry analysis for antigens: α-actin, vimentin, IGF-1, MMP-2, FGF-2, C-Myc, PSCA, AR, Erα and ERβ.

**Results:**

Reactive stroma with intense desmoplastic reactivity was significantly more frequent in intermediate (Gleason 7, 3+4) and high grade tumors (Gleason 7, 4+3). The group with intense stromal reactivity showed significant higher levels of Vimentin, IGF-1, MMP-2, FGF-2, C-Myc, PSCA and ERα.

**Conclusions:**

It can be concluded that RStr may be a predictive marker of Pca progression, since it was associated with increase of growth factors, imbalance of androgen and estrogen receptors and presence of malign prostatic stem cells.

## INTRODUCTION

Prostatic epithelium comprises three cellular types: luminal or columnar, basal and neuroendocrine (1). Luminal epithelial cells are the most frequent cell type in normal and hyperplasic epithelium and represent the exocrine compartment of prostate (2). Since tumor cells express similar characteristics to luminal cells, mutant luminal cells were considered precursors of adenocarcinoma (3, 4). These cells express androgen receptor (AR) and respond to androgen and are androgen-dependent (5). On the other side, basal cells are relatively undifferentiated, independent of androgens but androgen-respondent, they do not show secretory activity and form the basal compartment of the prostate (6). The neuroendocrine cells do not respond to androgens (7–9) and can modify in prostate cancer (number, histology and function) with a suggestive regulatory role in that disease (10, 11).

Reactive stroma (RStr is defined as the microenvironment closely adjacent to epithelium able to coordinate several activities as wound repair, homeostasis changes and interaction with neoplastic complexes, comprising an dynamic environment that influence directly the behavior of epithelial cells and perform tissue repair after lesion (12). Modifications of peritumor stroma start in prostatic intraepithelial neoplasia (PIN) and include phenotypic alterations of stromal cells, remodeling of extracellular matrix and induction of angiogenesis (13, 14). Reactive stroma (RStr) is defined as a new stroma environment in response to carcinoma. It follows tumor growth and is characterized by an increase of inflammatory cells, desmoplastic reaction, increase of angiogenesis and growth factors, with remodeling of extracellular matrix (15). RStr has a fibroblastic and a myofibroblastic compound associated to tumor and the origin of these cells is not clearly understood. Some authors suggest that these cells are originated from the prostatic stroma or smooth muscle or even stem cells (14, 15). Stem cells have the capacity of self-renovation and regeneration throughout adult life and are present in the epithelial and stromal compartments (16).

At the prostate several biological processes (regulation of proliferation and cellular differentiation, mitogenic activity, secretory processes and tumor growth) are regulated and/or influenced by different growth factors, such as IGF (insulin growth factor), FGF (fibroblast growth factor), VEGF (vascular endothelium growth factor), transforming growth factors, metalloproteinases and PSCA (prostate stem cell antigen) (17–22).

Additionally, testosterone is an important stimulant to prostatic cell proliferation, mainly when it's more potent form di-hydro-testosterone (DHT) bind to androgen receptors of cells from the epithelial and stromal compartments (23, 24), so those above mentioned processes are under direct influence of androgens, estrogens and their alpha (ERα) and beta (ERβ) receptors (25, 26).

Neoplastic transformation consists of a multi-causal process, where normal controls of cellular proliferation and interaction cell-to-cell are lost. Aberrant activation of proto-oncogenes along with non-regulated inhibition of tumor suppressor genes are fundamental in that process. In that context, stand out proto-oncogene C-MYC. In tumors, the scarce vascularization and the high proliferative profile lead to a hypoxic status (known as Warburg effect) that is able to induce the expression of C-MYC, that promotes an energetic reinforcement through glycolysis and that can additionally act as a suppressor of antiangiogenic factors in an attempt to oppose hypoxia and to promote adequate metabolic supply demanded by the tumor (27).

In view of the facts discussed above, it is essential to establish a correlation between the stromal microenvironment of prostate adenocarcinoma through morphologic and molecular characterization, and also to determine any association of growth factors, matrix metalloproteinases, sexual hormone and stem cell receptors with tumors with worse prognosis.

## MATERIALS AND METHODS

### Human Samples and Histopathological Analysis of Reactive Stroma

Forty prostatic samples of patients submitted to retropubic radical prostatectomy with 60–80 years old (median 71 years) were collected. Samples were obtained from the collection of the Department of Pathology of the Hospital de Clínicas da Universidade Estadual de Campinas (UNICAMP).

Samples were collected from the peripheral region based on the division of the posterior side, with basal to apical orientation of the organ. Next, samples were fixed in 10% buffered formaldehyde for 12 hours. After fixation, tissue samples were routinely processed (inclusion in paraffin, 5μm sections and Hematoxilin-Eosin staining).

Pca diagnosis was based in morphological criteria and classified according to Gleason system by a senior pathologist of the Department of Pathology of the Faculdade de Ciências Médicas da Universidade Estadual de Campinas (UNICAMP).

For reactive stroma analysis, the prostatic samples were divided in two groups (20 samples per group): Group-1: PCa samples without reactive stroma (Grade-0), Group-2: Pca samples with intense stromal reactivity (Grade-3).

Stromal reactivity was determined at the Urogenital Carcinogenesis Laboratory and Immunotherapy of the Biological Institute of UNICAMP, using Masson Trichromic stain. The intensity of reactive stroma was evaluated by the frequency (in percentage) of smooth muscular fibers (stained red with Masson Trichromic) adjacent to neoplastic areas in each sample with an augment of x400. Images were captured by photomicroscope Leica DM2500 equipped with a Leica camera DFC295 and analyzed by the software Leica LAS V3.7 for image analysis. The percentage of smooth muscular fibers adjacent to neoplastic areas was graded and expressed as 0>50% of smooth muscular fibers adjacent to neoplastic ducts, 1:36–50% of smooth muscular fibers adjacent to neoplastic ducts, 2:15–35% of smooth muscular fibers adjacent to neoplastic ducts, 3: 0–14% of smooth muscular fibers adjacent to neoplastic ducts. For this study intermediate levels of reactive stroma (grades 1 and 2) were discarded.

The study was approved by the Ethical Committee of Faculdade de Ciências Médicas/UNICAMP (#0094.0146.000-08).

### Immunohistochemistry for antigens: α-actin, Vimentin, IGF-1, MMP-2, FGF-2, C-Myc, PSCA, AR, ERα and Erβ

The same prostatic samples of 40 patients used for histopathological analysis ere submitted to immunohistochemistry. Antigen recovery was obtained by incubating the slices in buffered citrate (pH 6.0) at 100oC in microwave. The blockage of endogenous peroxidase was obtained with H2O2 and posterior incubation with a blocking solution with bovine serum albumin (BSA) for 1 hour at room temperature. After that, the antigens were localized with specific antibodies (Table-1), diluted in BSA and incubated overnight at 4oC. It was used the MACH 4 Universal HRP-Polymer® (Biocare Medical) kit for antigen detection, according to the manufacture instructions. Posteriorly, the slices were revealed with diaminobenzidin (DAB), counter-stained with Harris Hematoxilin and evaluated at the photomicroscope.

**Table 1:** Characteristic of primary antibodies for immuno-staining.

Primary antibodies	Host species	Code	Soource
α-actin	Mouse (monoclonal)	sc-32251	Santa Cruz, Biotechnology, EUA
Vimentin	Mouse (monoclonal)	ab8069	Abcam, EUA
IGF-1	Rabbit (policlonal)	sc-720	Santa Cruz, Biotechnology, EUA
MMP-2	Mouse (monoclonal)	ab86607	Abcam, EUA
FGF-2	Rabbit (policlonal)	sc-79	Santa Cruz, Biotechnology, EUA
C-Myc	Rabbit (policlonal)	ab32072	Abcam, EUA
PSCA	Rabbit (policlonal)	251249	Abbiotec, EUA
AR	Rabbit (policlonal)	ab74272	Abcam, EUA
ERα	Rabbit (policlonal)	04-227	Merck-Millipore, EUA
ERβ	Mouse (monoclonal)	ab16813	Abcam, EUA

In order to evaluate the intensity of the antigen immunoreactions, the percentage of positive epithelial and/or stromal cells was examined in 10 fields for each antibody with an augment of 400x. The intensity of staining was grade in a 0–3 scale and expressed as 0 (no immunoreactivity), 0% of positive epithelial and/or stromal cells, 1 (weak immunoreactivity), 1–35% of positive epithelial and/or stromal cells, 2 (moderate immuno-reactivity), 36–70% of positive epithelial and/or stromal cells, 3 (intense immunoreactivity), >70% of positive epithelial and/or stromal cells.

### Statistical analysis

The histopathological and immunohistochemistry analysis for different antigens were evaluated with the proportion test. For these analyses, an error type-I of 5% was considered statistically significant.

## RESULTS

### Histopathological Analysis of Reactive Stroma

**Figure 1:**
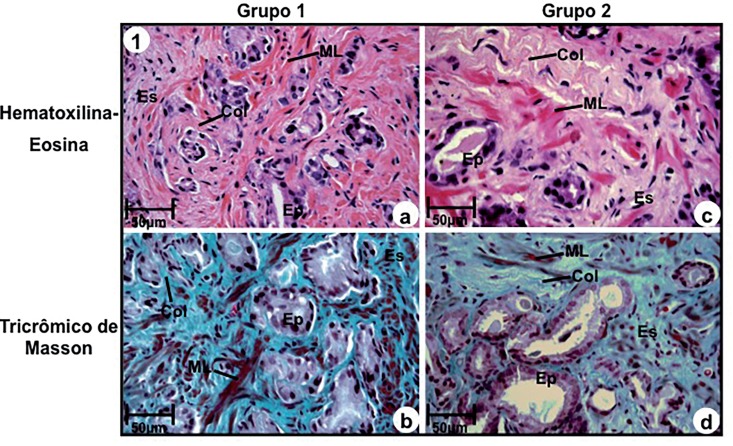
Photomicrography of prostatic peripheral zone of groups 1 (a, b) and 2 (c, d). (a) and (b) Stroma without desmoplastic reaction consisting of excess of smooth muscular fibers (ML) and thin collagen fibers (col) adjacent to prostatic ducts. Stains: Hematoxilin-Eosin (a) and Masson Trichromic (b). (c) and (d) Stroma with intense desmoplastic reactivity consisting of excess of collagen fibers (Col) and rare smooth muscular fibers (ML), stains: Hematoxillin-Eosin (c) and Masson Trichromic (d). **a-d: Ep=**secretory epithelium; Es-stroma. Scale of 50μm.

Stroma without desmoplastic reaction (Group-1) was characterized by the presence of a great amount of smooth muscular fibers, above 50% of adjacent ducts with collagen fibers interspersed among the smooth muscular fibers (Figures 1a and 1b).

In relation to stroma with intense desmoplastic reaction (Group-2), it was characterized by an outstanding increase and thickening of collagen fibers, associated to an intense reduction (below 14%) of smooth muscular fibers (Figures 1c and 1d). Stroma without desmoplastic reaction (Group-1) was significantly more frequent in Gleason 4 (2+2), 5 (3+2) and 6 (3+3) (Table-2), and absent in high grade tumors (Gleason 7, 4+3) (Table-2).

**Table 2:** Distribution of gleason score and stromal reactivity in prostatic adenocarcinoma without stromal reactivity (group-1) and with intense stromal reactivity (group-2)

Gleason score	Number of cases (%)	Group-1	Group-2
Gleason 4 (2+2)	1 (2.5%)	1 (100.0%)*	0 (0.0%)
Gleason 5 (3+2)	2 (5.0%)	2 (100.0%)*	0 (0.0%)
Gleason 6 (3+3)	19 (47.5%)	16 (84.2%)*	3 (15.8%)
Gleason 7 (3+4)	10 (25.0%)	1 (10.0%)	9 (90.0%)*
Gleason 7 (4+3)	8 (20.0%)	0 (0.0%)	8 (100.0%)*
**Tot**al	**40 (100.0%)**	**20 (50.0%)**	**20 (50.0%)**

Stroma with intense desmoplastic reactivity was significantly more frequent in intermediate (Gleason 7, 3+4), and high grade tumors (Gleason 7, 4+3) and in low grade tumors it was observed in only 3 cases with Gleason 6 (3+3) (Table-2).

### Immunohistochemistry of antigens: α-actin, Vimentin, IGF-1, MMP-2, FGF-2, C-Myc, PSCA, AR, ERα and ERβ

**Figure 2:**
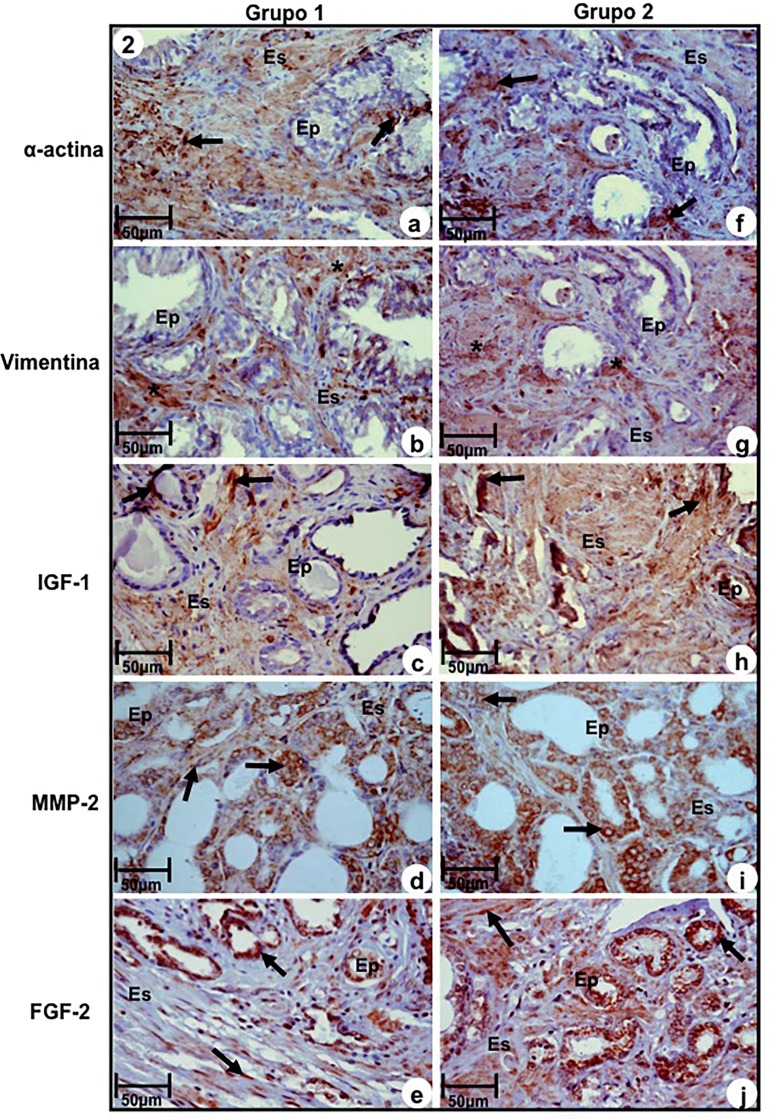
Immuno-staining of antigens α-actin, Vimentin, IGF-1, MMP-2 and FGF-2 at prostatic peripheral zone of Groups-1 (a, b, c, d, e) and 2 (f, g, h, i, j). (a) and (f) Immunoreactivity to α-actin (arrows). (b) and (g) Immunoreactivity to Vimentin (asterisks) in myofibroblasts. (c) and (h) Imunoreactivity to IGF-1 (arrows) in epithelium and stromal compartments. (d) and (i) Immunoreactivity to MMP-2 (arrows) in epithelial and stromal compartments. (e) and (j) Immunoreactivity to FGF-2 (arrows) in cells of secretory epithelium and fibroblasts of stromal compartment. **a-j, Ep–**secretory epithelium; **Es-**stroma. Scale of 50μm.

Immunoreactivity to α-actin, a marker of smooth muscle, was significantly more intense in Group-1 in relation to Group-2. The last one showed moderate immune staining (Figures 2a and 2f, Table-3). On the contrary, immunoreactivity of vimentin, a fibroblast and myofibroblast marker, was significantly more intense in Group-2 in relation to Group-1, that showed moderate immunoreactivity (Figures 2b and 2g, Table-3).

**Table 3:** Intensity of immuno-staining of different antigens in epithelial and stromal cells of prostatic adenocarcinomas without stromal reactivity (group-1) and with intense stromal reactivity (group-2).

		Groups
Antigens	Group 1 (n=20)	Group 2 (n=20)
α-actin	3 (80.3%)*	2 (69.0%)
Vimentin	2 (61.4%)	3 (92.4%)*
IGF-1	2 (57.6%)	3 (96.8%)*
MMP-2	2 (67.3%)	3 (89.7%)*
FGF-2	2 (62.7%)	3 (91.5%)*
C-Myc	2 (65.5%)	3 (93.3%)*
PSCA	2 (56.5%)	3 (85.8%)*
AR	1 (30.9%)	2 (59.5%)*
Erα	2 (39.7%)	3 (77.9%)*
Erβ	2 (38.6%)*	1 (26.5%)

**0** (absence of immunoreactivity), 0% of positive epithelial and/or stromal cells; **1** (weak immunoreactivity), 1–35% of positive epithelial and/or stromal cells; **2** (moderate immunoreactivity), 36–70% of positive epithelial and/or stromal cells; **3** (intense immunoreactivity), >70% of positive epithelial and/or stromal cells.

Immunoreaction to IGF-1, MMP-2 and FGF-2 were significantly more intense in epithelium and stroma of samples of Group-2, compared to Group-1, that showed moderate reactivity (Figures 2c, 2d, 2e, 2h, 2i, and 2, Table-3).

**Figure 3:**
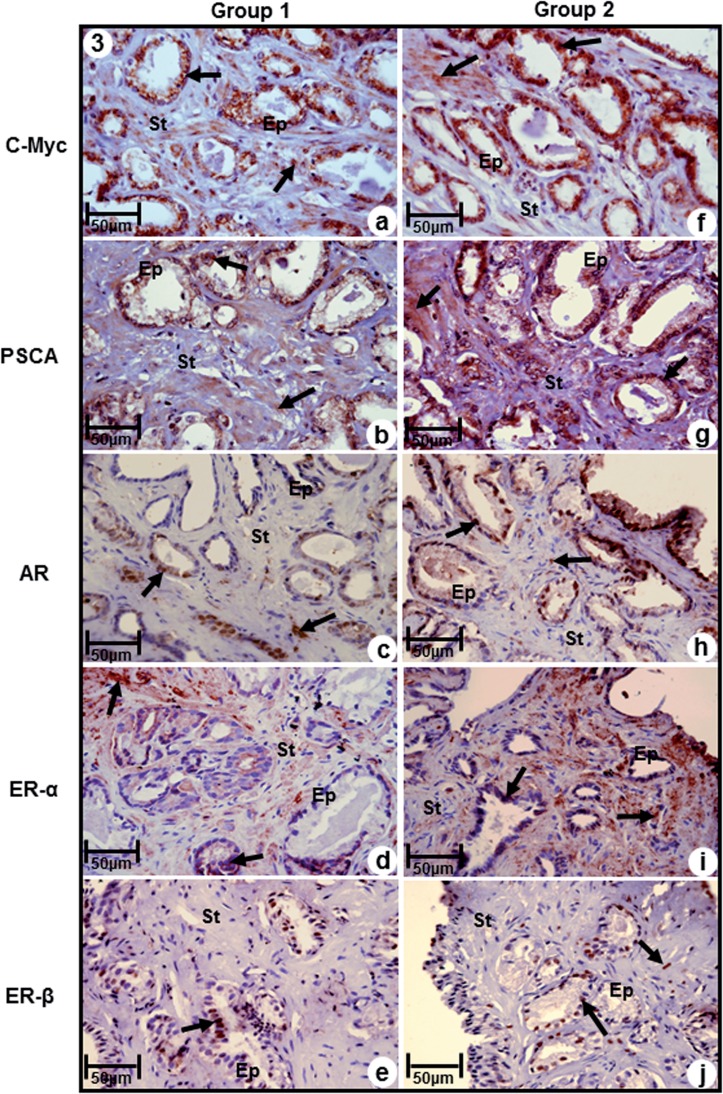
Immuno-staining of antigens C-Myc, PSCA, AR, ERα and ERβ in prostatic peripheral zone of Groups 1 (a, b, c, d, e) and 2 (f, g, h, i, j). (a) and (f) Immunoreactivity to C-Myc (arrows) in epithelial and stromal compartments (b) and (g) Immunoreactivity to PSCA (arrows)) in epithelial and stromal compartments (c) and (h) Immunoreactivity to AR (arrows)) in epithelial and stromal compartments (d) and (i) Immunoreactivity to ERα (arrows)) in epithelial and stromal compartments (e) and (j) Immunoreactivity to ERβ (arrows)) in epithelial and stromal compartments. **a-j, Ep=**secretory epithelium; **Es=**stroma. Scale of 50μm.

Immunoreactivity to C-Myc was significantly more intense in both epithelium and stroma in samples of Group-2, and moderate in Group-1 (Figures 3a. and 3f, Table-3). Likewise, immuno-reactivity to prostatic stem cell antigen (PSCA) was observed both in epithelial and stromal compartments, being significantly more intense in samples of Group-2, compared to Group-1, that showed moderate immunoreactivity (Figures 3b and 3g, Table-3).

Immunoreactivity for AR was moderate in both epithelium and stroma of samples of Group-2, while in Group-1 it was observed low immunoreactivity (Figures 3c and 3h, Table-3). Immunoreactivity to ERα was predominant in stromal compartment in both groups, being intense in Group-2 and moderate in Group-1 (Figures 3d and 3i, Table-3).

In contrast, immunoreactivity for Erβ was predominant in epithelial compartment of both groups, and immune staining of stroma was present only in Group-2 (Figures 3e and 3j). Immunoreactivity of this marker was moderate in Group-1 and weak in Group-2 (Figures 3e and 3, Table-3).

## DISCUSSION AND CONCLUSIONS

Interaction of epithelium-stroma has a primary role in maintenance of structure and functioning of prostate. Stromal cells associated to tumor cells respond to androgens forming growth factors that lead to interruption of epithelium-stroma homeostasis, initiating growth and migration processes, angiogenesis, apoptosis and tumor metastasis (28).

In the present study RStr was morphologically characterized by the significant reduction of smooth muscular fibers and excess of collagen fibers in stroma adjacent to neoplastic ducts. Intense stromal reactivity was observed in intermediate (Gleason 7, 3+4) and high grade tumors (Gleason 7, 4+3) and in low grade tumors it was observed in only 3 cases with Gleason 6 (3+3), pointing out that RStr may be considered a predictive marker of tumor progression.

In relation to molecular characterization of RStr, the results showed increased reactivity to vimentin, IGF-1, MMP-2, FGF-2 and C-Myc in samples with intense stromal reactivity when compared to samples without reactivity. Such markers were fundamental to activation of RSTr and made the prostatic microenvironment favorable to tumor progression due to increase of imbalance of epithelium-stroma interaction.

Several studies have demonstrated that RStr is associated with lower survival free of disease. Yanagisawa (29) analyzed prostatic biopsies of 205 patients and demonstrated a significant difference between high and low reactive RSTr and concluded that the intensity of RStr may be considered a prognostic factor independent of biochemical recurrence. Also, Ayala (15), after analyzing samples from radical prostatectomy and Billis (30), that analyzed 266 needle prostatic biopsies showed that RStr could only be considered an prognostic factor independent of biochemical recurrence when it showed intense stromal reactivity. Still, RStr with intense stromal reactivity was observed in Ayala (15), Yanagisawa (29) and Billis (30) studies in 9.0%, 6.7% and 5.3% of samples respectively, with very similar frequencies among the studies. However, RStr with low stromal reactivity frequencies were very distinct among these three papers: 6.25% (Ayala, 15), 0.5% (Yanagisawa, 29) and 53.8% (Billis, 30). That reflects that lack of uniform morphological criteria to characterize RSTr.

In conclusion, the present study shows a new approach to Pca diagnosis and the results demonstrated RStr may be considered a predictive marker of PCa progression, since increase of vimentin, IGF-1, MMP-2, FGF-2 and C-MTC are evidences of worse tumor prognosis and the occurrence of prostatic stem cells (elevation of PSCA) and the balance of AR and Erα with concurrent inhibitory action of ERβ at RStr point to a greater malignancy of these tumors as well as an indication of recurrence. However, new studies are necessary to better understanding of this microenvironment and upgrading of available treatments of prostate cancer, and the development of new modalities that assure better clinical results and quality of life of patients.

## Abbreviations

AR =: androgen receptor
EstR =: Reactive stroma
IGF =: Growth factor Homologous Insulin
FGF =: Fibroblast Growth Factors
VEGF =: Vascular Endothelial Growth Factor
MMP =: Matrix metalloproteinase
CaP =: Prostate cancer
PSCA =: Antigen for Prostate Stem Cell
DHT =: dihydrotestosterone
ERα =: Estrogen Receptor Alpha
ERβ =: Estrogen Receptor Beta
